# Editorial: Case reports in veterinary neurology and neurosurgery

**DOI:** 10.3389/fvets.2025.1614212

**Published:** 2025-05-20

**Authors:** Koen M. Santifort, Joao Miguel De Frias, Simone Spinillo, Bruno A. Lopes, Susana Monforte Monteiro, Andrea Tipold

**Affiliations:** ^1^IVC Evidensia Small Animal Referral Hospital Arnhem, Neurology, Arnhem, Netherlands; ^2^IVC Evidensia Small Animal Referral Hospital Hart van Brabant, Neurology, Waalwijk, Netherlands; ^3^Hospital for Small Animals, Royal (Dick) School of Veterinary Studies, The University of Edinburgh, Edinburgh, United Kingdom; ^4^ChesterGates Veterinary Specialists, Chester, United Kingdom; ^5^Southfields Veterinary Specialists, Linnaeus Veterinary Limited, Basildon, United Kingdom; ^6^Department of Veterinary Medicine, University of Cambridge, Cambridge, United Kingdom; ^7^Department of Small Animal Medicine and Surgery, University of Veterinary Medicine, Hannover, Germany

**Keywords:** neurology, neuropathology, neurosurgery, neuro-oncology, case series

Case reports have long been instrumental in the progression of medical knowledge, offering detailed insights into unique clinical scenarios that enhance our understanding of disease processes, diagnostic challenges, and therapeutic strategies. In the realm of veterinary neurology and neurosurgery, such reports are invaluable for documenting rare conditions, innovative treatments, and unexpected outcomes across a diverse range of animal species. The aim of this “*Case reports in veterinary neurology and neurosurgery***—***volume I*” Research Topic was and that of the volumes to come remains to be to establish a lasting and prominently featured platform for the publication of case reports in veterinary neurology and neurosurgery, fostering a collaborative environment for knowledge sharing and professional development. By sharing case-based clinical experiences and outcomes, veterinarians contribute to a collective database of knowledge that benefits the entire profession. This collaborative effort can contribute to advances in the field of veterinary neurology as a whole. Case reports (single cases) as well as case series (multiple cases) are included in this Research Topic.

This first Volume of “*Case reports in veterinary neurology and neurosurgery*” contains 17 publications. Their strengths and learning points are highlighted and could contribute to clinical management and potentially spark future, larger prospective studies. The case reports of Volume 1 describe features in the elderly, but also in young dogs, from clinical phenomena to pathophysiology, and different neurosurgery techniques to neuropathology. Known and previously unknown genetic mutations and their clinical relevance, new aspects in inflammatory central nervous systems diseases, and insights for particular neoplastic disorders are discussed.

Exploring the genetic underpinnings of limb length variations in a family of Dachshunds, Sullivan et al. contributed to the understanding of chondrodysplasia and its genetic markers with their report titled “*FGF4L1 retrogene insertion is lacking in the tall dachshund phenotype*”. This manuscript highlights how the limb length is important for this breed to maintain working ability, aiming to help in aspects of animal welfare protection as this breed has been banned recently from exhibition in Germany. Eguchi et al. identified and documented a novel genetic mutation linked to hereditary myotonia in their case report titled “*A CLCN1 complex variant mutation in exon 15 in a mixed-breed dog with hereditary myotonia*”, enhancing the genetic profiling of neuromuscular disorders in canines.

Kang et al., in their case report titled “*Ischemic brain infarction and cognitive dysfunction syndrome in an aged dog*”, highlighted a possible correlation between ischemic brain infarction and cognitive dysfunction in geriatric canines, emphasizing the importance of considering vascular events in behavioral changes.

In a case report by Jackson et al., titled “*Presumed cerebral salt wasting syndrome in a 10-week-old German Shorthaired Pointer*”, this particular syndrome following traumatic brain injury in a dog is described, enlightening the reader about this unusual condition, as well as describing a new diagnostic method that could be used in similar presentations.

The case report titled “*Positioning head tilt observed in a dog and four cats with bilateral peripheral vestibular dysfunction*” by Tamura et al. documented a unique clinical sign associated with bilateral vestibular dysfunction, aiding in the recognition and diagnosis of these conditions.

Yin et al. provided valuable insights into the clinical presentation, diagnostic challenges, histological characteristics, and management strategies for intraneural perineuriomas in canines in their report titled “*Intraneural perineurioma in dogs: a case series and brief literature review*”.

Rancilio et al. have, in their report titled “*Suprasellar and trigeminal nerve oligodendroglioma with pseudoprogression after radiotherapy and serial MRI in a dog*”, detailed the phenomenon of pseudoprogression and cranial nerve involvement in a canine oligodendroglioma. This phenomenon is well-described in the human counterpart, but its occurrence in dogs has been rarely reported, solidifying the use of canine patients as a good comparative model of human neuro-oncologic conditions.

A surgical approach for resecting a fourth ventricular meningioma in a dog is described by Jeong et al. in the case report titled “*Gross total resection of a primary fourth ventricular meningioma using the telovelar approach in a dog*”, providing a reference for similar neurosurgical procedures.

Demonstrating the effectiveness of compartmental resection in treating malignant nerve sheath tumors, Smith et al. in their case report titled “*Resolution of lameness via compartmental resection of a malignant nerve sheath neoplasm of the median nerve in a dog*” highlighted the relevance of considering neurogenic causes in dogs presenting with lameness.

Hobert et al. in their case report titled “*One-stage craniectomy and cranioplasty digital workflow for three-dimensional printed polyetheretherketone implant for an extensive skull multilobular osteochondosarcoma in a dog*”, showcased the application of 3D printing technology in creating custom implants for cranial reconstruction, advancing surgical techniques in veterinary oncology and neurosurgery.

The case report titled “*Intracranial epidermoid cyst in a cat*” by Terao et al. included information on the diagnosis and management of a rare diagnosis in a cat, contributing to the knowledge of diagnostic features of such a case.

The feasibility and outcomes of surgically removing rare intracranial dermoid cysts in a feline patient is demonstrated by the case report titled “*Successful surgical resection of an intracranial frontal lobe dermoid cyst in a cat*” by Nakano et al., contributing to surgical approaches in similar cases.

Shi et al. in their report titled “*Endoscope-assisted single-incision double-channel mini-open hemilaminectomy for the treatment of acute thoracolumbar intervertebral disc disease in 11 dogs*” described a minimally invasive surgical technique for thoracolumbar disc disease, emphasizing its efficacy and potential benefits over traditional methods.

In the report titled “*Surgical management of single-level thoracolumbar vertebral body segmentation and formation failure causing progressive thoracolumbar myelopathy in three adult large-breed dogs*”, Couto et al. provided insights into surgical interventions for congenital vertebral malformations causing myelopathy, offering an innovative way to manage such anomalies.

In the report titled “*Double adjacent ventral slot in two medium-sized breed dogs*”, Cojocaru highlighted a modified surgical technique addressing cervical disc disease, offering an approach for more extensive ventral decompression.

Santifort et al. in their case report titled “*Necrotizing leukomyelitis and meningitis in a Pomeranian*”, presented a case of clinically severe inflammatory spinal cord disease of unknown etiology, underscoring the need for inclusion of this differential diagnosis, based on clinical findings as well as MRI findings.

Last but not least, in light of a well-known risk in human MRI studies, the report titled “*Radiofrequency-induced thermal burn injury in a dog after magnetic resonance imaging*” notified the veterinary profession of the possibility of this complication of veterinary MRI procedures, underscoring the need for caution and monitoring during and after imaging (Lichtenauer et al.).

In summary, all these case reports offered contributions to our collective knowledge concerning various topics, including surgical approaches and techniques, differential diagnosis, medical and alternative treatment, pathophysiology, genetics, clinical management, and clinical phenomenology. Through their respective contributions, we continue to learn from single case reports and case series, opening new avenues for research and generating clinically useful insights.

